# Linagliptin and Vitamin D3 Synergistically Rescue Testicular Steroidogenesis and Spermatogenesis in Cisplatin-Exposed Rats: The Crosstalk of Endoplasmic Reticulum Stress with NF-κB/iNOS Activation

**DOI:** 10.3390/molecules27217299

**Published:** 2022-10-27

**Authors:** Rania A. Elrashidy, Esraa M. Zakaria, Asmaa M. Elmaghraby, Rasha E. M. Abd El Aziz, Ranya M. Abdelgalil, Rehab M. Megahed, Asmaa A. Elshiech, Doaa E. A. Salama, Samah E. Ibrahim

**Affiliations:** 1 Biochemistry Department, Faculty of Pharmacy, Zagazig University, Zagazig 44519, Egypt; 2 Pharmacology Department, Faculty of Pharmacy, Zagazig University, Zagazig 44519, Egypt; 3 Histology Department, Faculty of Medicine for Girls, Al-Azhar University, Cairo 11651, Egypt; 4 Biochemistry Department, Faculty of Medicine for Girls, Al-Azhar University, Cairo 11651, Egypt; 5 Anatomy and Embryology Department, Faculty of Medicine for Girls, Al-Azhar University, Cairo 11651, Egypt; 6 Forensic Medicine and Clinical Toxicology Department, Faculty of Medicine for Girls, Al-Azhar University, Cairo 11651, Egypt; 7 Pathology Department, Faculty of Medicine for Girls, Al-Azhar University, Cairo 11651, Egypt; 8 Pathology Department, School of Medicine, Badr University, Cairo 11829, Egypt; 9 Physiology Department, Faculty of Medicine for Girls, Al-Azhar University, Cairo 11651, Egypt

**Keywords:** cisplatin, endoplasmic reticulum stress, linagliptin, steroidogenesis, vitamin D3

## Abstract

This study investigated the therapeutic effect of linagliptin and/or vitamin D3 on testicular steroidogenesis and spermatogenesis in cisplatin-exposed rats including their impact on endoplasmic reticulum (ER) stress and NF-κB/iNOS crosstalk. Cisplatin (7 mg/kg, IP) was injected into adult male albino rats which then were orally treated with drug vehicle, linagliptin (3 mg/kg/day), vitamin D3 (10 μg/kg/day) or both drugs for four weeks. Age-matched rats were used as the control group. Serum samples and testes were collected for further analyses. Cisplatin induced testicular weight loss, deteriorated testicular architecture, loss of germ cells and declined serum and intra-testicular testosterone levels, compared to the control group. There was down-regulation of steroidogenic markers including StAR, CYP11A1, HSD3b and HSD17b in cisplatin-exposed rats, compared with controls. Cisplatin-exposed rats showed up-regulation of ER stress markers in testicular tissue along with increased expression of NF-κB and iNOS in spermatogenic and Leydig cells. These perturbations were almost reversed by vitamin D3 or linagliptin. The combined therapy exerted a more remarkable effect on testicular dysfunction than either monotherapy. These findings suggest a novel therapeutic application for linagliptin combined with vitamin D3 to restore testicular architecture, aberrant steroidogenesis and spermatogenesis after cisplatin exposure. These effects may be attributed to suppression of ER stress and NF-kB/iNOS.

## 1. Introduction

Steroidogenesis entails a multi-step process by which cholesterol is converted into biologically active steroid hormones. In the adult testis, steroidogenesis occurs in Leydig cells for testosterone production in response to luteinizing hormone (LH) secretion by the pituitary gland. The process of steroidogenesis comprises two major enzyme groups: cytochrome P450 (CYPs) and hydroxysteroid dehydrogenases (HSDs). These enzymes are found on the inner mitochondrial and endoplasmic reticulum (ER) membranes of Leydig cells. Steroidogenesis begins with trafficking of cholesterol by steroidogenic acute regulatory protein (StAR) from the outer to the inner mitochondrial membrane, where the enzyme cytochrome P450, family 11, subfamily A, polypeptide 1 (CYP11A1) converts cholesterol to pregnenolone. The resulting pregnenolone is further metabolized to testosterone by the action of three enzymes in the ER membranes, namely 3 beta-hydroxysteroid dehydrogenase (HSD3b), CYP17A1 and17-beta-hydroxysteroid dehydrogenase (HSD17b) [1].

Testosterone is essential for spermatogenesis and the maintenance of secondary sexual functions. Deficiency of testosterone in adult males impairs spermatogenesis as well as fertility and can lead to health consequences including increased body fat, suppressed cognitive function, and decreased muscle mass and bone density [2]. Several studies have reported that the steroidogenic function of Leydig cells is worsened by wide range of factors including aging, hormonal disrupters and chemotherapy [3]. Cisplatin, cis-diamminedichloroplatinum (II), is a platinum-derived chemotherapeutic agent extensively used to treat a range of solid tumors worldwide. Despite its promising results on cancer and survival rate, cisplatin-based chemotherapy is coupled with toxic side effects on male reproductive health. Cisplatin has been shown to induce Leydig cell dysfunction, reduce testosterone levels and disrupt spermatogenesis. The toxic effects of cisplatin also extend to accessory sex organs such as epididymis and vas deferens, thereby affecting sperm maturation process [4]. Therefore, developing new therapies for preserving the fertility in male cancer survivors following cisplatin therapy is potentially important.

In eukaryotic cells, the ER is the major intracellular organelle responsible for synthesis, folding, modification and transport of proteins. The accumulation of misfolded and unfolded proteins in the ER lumen eventually disrupts ER homeostasis causing ER stress. The ER stress triggers the unfolded protein response (UPR), mediated by three major sensors: protein kinase RNA-like ER kinase (PERK), inositol requiring protein 1 (IRE1) and activating transcription factor 6 (ATF6) [5]. Accumulating evidence has linked the ER stress to impaired testicular steroidogenesis and male infertility. Hypoxia-induced ER stress has been reported to suppress the expression of StAR and HSD3b along with a decline in the testosterone synthesis [6]. ER stress has been found to underlie the aging-related steroidogenic dysfunction and reduction in testosterone secretion [7,8]. It has become apparent that ER-stress is coupled with the activation of inflammatory pathways mediated by nuclear factor kappa light chain enhancer of activated B cells (NF-κB), NLRP3 inflammasome and inducible nitric oxide synthase (iNOS) [9]. Persistent ER stress can also amplify the apoptotic signaling through transcription factor C/EBP homologous protein (CHOP), which consequently mediates a programmed cell death [10]. Accordingly, the crosstalk between ER stress and NF-κB becomes an emerging area of interest in the context of testicular dysfunction. On the other hand, cisplatin has been shown to induce ER stress in the testicular tissue of rats [11,12]; however, whether cisplatin-related ER stress and NF-κB/iNOS activation could disrupt testosterone biosynthesis and spermatogenesis remain unresolved.

The active form of vitamin D3, 1α, 25 dihydroxy cholecalciferol, regulates calcium and phosphorus metabolism through binding to its receptor, vitamin D receptor (VDR). Increasing evidence indicates that VDR is widely expressed in the testes, vas deferens, and spermatozoa, suggesting a potential role of vitamin D3 in the gonadal axis, steroidogenesis and reproductive health as a whole, beyond its role in bone metabolism [13]. A previous study demonstrates that vitamin D3 deficiency can adversely impact sex hormone levels as well as sperm quality and quantity, which ultimately leads to male infertility [14].

Linagliptin is a selective dipeptidyl peptidase-4 (DPP-4) inhibitor approved as an oral anti-diabetic drug. Linagliptin has been shown to exert various pleiotropic effects, through incretin-independent mechanisms [15]. Recently, we showed that linagliptin treatment can increase the circulatory and testicular levels of testosterone in cisplatin-treated rats, suggesting a favorable effect of linagliptin on testicular steroidogenesis [16]; however, the precise underlying mechanisms are not fully explored. In this context, the present study was designed to investigate the effects of vitamin D3 and linagliptin therapies either individually or in combination on steroidogenesis and spermatogenesis in cisplatin-exposed rats, with particular focus on their roles on the interaction between ER stress and NF-κB/iNOS activation.

## 2. Material and Methods

### 2.1. Drugs and Chemicals

Cisplatin was obtained from ASTA Medical (Frankfurt am Main, Germany). Linagliptin (Trajenta^®^) was purchased from Boehringer Ingelheim Roxane Inc. (Columbus, OH, USA). Vitamin D3 (Devarol^®^ 200.000 IU/2 mL, 5 mg/2 mL) was supplied from Chemipharm Pharmaceuticals Industries (Cairo, Egypt). Other chemicals for biochemical assays were obtained from Sigma-Aldrich (St. Louis, MO, USA).

### 2.2. Animals and Experimental Design

Fifty male Wistar albino rats (10 weeks old) weighing 220–240 g were purchased from the animal facility of Zagazig University. Rats were maintained under standard laboratory conditions of 12 h light/dark cycle and room temperature 22–24 °C in plastic cages with free access to food and water. The animals were handled according to the guidelines of the National Institutes of Health (NIH) for animal research. All the experimental protocols were approved by the institutional ethical committee of animal research.

Rats were acclimatized for one week prior to experimental use then randomly assigned into five groups (*n* = 10), as follows: (I) Control group (CON): received a single intraperitoneal (i.p.) injection of saline on the first day, then given matched volumes of drug vehicles, for four weeks. (II) Cisplatin-exposed group (CIS): received a single i.p. injection of cisplatin (7 mg/kg) then was given matched volumes of drug vehicles, for four weeks. (III) Vitamin D3-treated group (VD3): received a single i.p. injection of cisplatin (7 mg/kg) then was orally treated with vitamin D3 (10 μg/kg/day, it was dissolved in corn oil) for four weeks. (IV) Linagliptin-treated group (LINA): received a single i.p. injection of cisplatin (7 mg/kg) then was orally treated with linagliptin (3 mg/kg/day, it was dissolved in distilled water), for four weeks. (V) Combined therapy group (VD3+LINA): received a single i.p. injection of cisplatin (7 mg/kg) then was treated with combination of linagliptin and vitamin D3 in the same dose levels, for four weeks. The selected dose of cisplatin has been shown to be optimal to induce testicular toxicity without excessive mortality in rats as reported previously [17,18]. The doses of linagliptin and vitamin D3 were chosen based on previous studies [16,19], respectively, and to be equivalent to the therapeutic doses used in clinical practice. Notably, one rat from the CIS group died throughout the study while all the other rats survived until the end of the treatment. At the end of the treatment period, rats were weighed then anaesthetized with thiopental sodium (30 mg/kg, i.p.). Blood was collected via heart puncture, allowed to coagulate for 20 min, centrifuged at 3500 rpm for 15 min for serum separation. Shortly before euthanasia, the testes were harvested and weighed. One testis was immediately fixed in 10% formaldehyde for histological analysis. The other testis was snap frozen in liquid nitrogen and stored at −20 °C for subsequent analyses.

### 2.3. Hormonal Analysis

Serum and testicular testosterone levels as well as serum LH levels were measured by commercially available rat enzyme linked immunosorbent assay (ELISA) kits (Enzo life sciences, San Diego, CA, USA), following the manufacturers’ instructions.

### 2.4. Measurement of Proinflammatory Cytokines

Levels of tumor necrosis factor-alpha (TNF-α) and interleukin-1beta (IL-1β) in testicular tissue homogenates were measured using commercially available rat ELISA kits (MyBioSource, San Diego, CA, USA), according to the manufacturer’s guidelines.

### 2.5. Quantitative Real-Time Polymerase Chain Reaction (qRT-PCR) Analysis

The relative gene expression of steroidogenic markers was evaluated by qRT-PCR. Briefly, total RNA was extracted from testicular tissue using TRIzol reagent (Invitrogen, Carlsbad, CA, USA). After determining the concentration and purity of the RNA, reverse transcription was performed using a complementary DNA reverse transcription kit (Invitrogen), following the manufacturer’s instructions. cDNA was then amplified by PCR in a real-time PCR thermocycler (Bio-Rad, Hercules, CA, USA) using SYBR Green Master Mix (2×) (Takara, Takara Bio Inc, Japan) and the following primer sequences: For **StAR**, forward primer: 5′-AGC AAG GAG AGG AAG CTA TGC-3′ and reverse primer: 5′-GGC ACC ACC TTA CTT AGC ACT-3′; For **CYP11A1**, forward primer: 5′-ACT TCC TGA GGG AGA ACG GC-3′ and reverse primer: 5′-TCC ATG TTG CCC AGC TTC TCC-3′; For **HSD3b**, forward primer: 5′-GAC AGG AGC AGG AGG GTT TGTGG-3′ and reverse primer: 5′-CTC CTT CTA ACA TTG TCA CCT TGG CCT-3′; For **HSD17b**, forward primer: 5′-CTG CTT GTG TGC CTC GTT TG-3′ and reverse primer: 5′-ACT GCC CAT TGT CCC ATT GA-3′. The reaction conditions were as follows: 1 cycle of activation and denaturation (10 min at 95 °C), followed by 40 cycles of denaturation (15 s at 95 °C), annealing and elongation (1 min at 60 °C). The mRNA expression of the target genes was quantified using the cycle threshold (2^−ΔΔCt^) method, relative to that of glyceraldehyde-3-phosphate dehydrogenase (GAPDH), as an internal control.

### 2.6. Measurement of ER Stress Markers

Frozen testicular tissue was homogenized in ice-cooled buffer phosphate saline (BPS) then centrifuged at 5000× *g* for 5 min. The supernatant was collected for assaying levels of PERK (LifeSpan BioSciences, Seattle, WA, USA; Catalog # LS-F16178) and CHOP (MyBioSource, San Diego, CA, USA; Catalog # MBS1607940) using commercially available rat ELISA kits, according to the supplier’s guidelines.

### 2.7. Histological Analysis

Specimens of testes were fixed in 10% formaldehyde, embedded in paraffin and then sectioned as 5 μm thick. After being deparaffinized, slices were stained with hematoxylin and eosin (H&E) for assessment of the general testicular architecture, periodic acid schiff (PAS) with hematoxylin counterstaining for the detection of basement membrane of the seminiferous tubules, and Sirius Red stain for detection of collagen fibers according to the standard protocols. Slides were examined using light microscopy (Primo star, ZEISS, Shanghai, China) and photomicrographs were taken using an Axiocam ERc 5s camera (ZEISS, Shanghai, China) at Histology Department, Faculty of Medicine for Girls, Al Azhar University.

To assess spermatogenesis, seminiferous tubules in H&E-stained sections were graded using Johnsen’s score (JS) from 1 to 10 according to tissue maturation and spermatogenesis status, as previously reported [20]. JS was determined in 20 randomly selected tubules under ×40 magnification and averaged for each section. Four sections from four different rats were analyzed in each group by a histologist in a blind manner. The scoring criteria were as follows: 1: tubular sclerosis; 2: only Sertoli cells without germinal cells; 3: only spermatogonia; 4: few spermatocytes but no spermatozoa or spermatids; 5: many spermatocytes but no spermatozoa or spermatids; 6: few early spermatids but no spermatozoa or late spermatids; 7: many early spermatids but no spermatozoa or late spermatids; 8: few late spermatids and less than five spermatozoa per tubule; 9: many late spermatids, slightly impaired spermatogenesis and disorganized epithelium; and 10: full spermatogenesis.

The count of Leydig and Sertoli cells were examined in H&E-stained slides using a light microscope at a magnification of ×400. The numbers of Leydig cells were counted in 20 random intertubular regions (an area surrounded by three seminiferous tubules), while Sertoli cells were counted in 20 seminiferous tubules per slide. Four testicular slides from four different rats were analyzed in each group by a histologist in a blind manner [16].

### 2.8. Morphometric Analysis

The morphometric analysis was performed using Image J software (1.51, National Institute of Health, Bethesda, MD, USA). A picture of known distance in micrometer was used for setting scale and conversion of values from pixels to micrometers. The mean diameter of seminiferous tubule and the epithelium thickness of the seminiferous tubules (from the basement membrane to lumen) were measured in 10 seminiferous tubules per slide in H&E-stained sections at ×10 magnifications. The seminiferous tubules that were round or close to round were randomly selected for measurement. The diameter of the seminiferous tubule was measured across the minor and major axes, and the mean diameter obtained. The results were expressed in μm. The thickness of basement membrane of seminiferous tubules was measured in 10 randomly selected fields per slide in PAS-stained sections at ×20 magnification. The results were also expressed in μm. The area% of the stained collagen fibers was measured in 10 randomly selected fields per slide in Sirius red-stained sections at ×20 magnification. The results were expressed as mean area% of collagen/μm^2^. All the morphometric analyses were performed in four slides from different animals per group by a histologist in a blind fashion [21].

### 2.9. Immunohistochemical Staining

The expressions of NF-κB p65 and iNOS were assessed by immunohistochemistry using the streptavidin-biotin complex method, as previously described [22,23]. After the antigen retrieval procedure using 0.01 M citrate buffer, the sections were incubated with 3% H_2_O_2_ and then incubated overnight at 4 °C with primary antibodies against NF-kB p65 or iNOS (Cat #ab16502, and #ab15323, respectively; Abcam, Cambridge, UK; dilution 1:100). All sections were then counterstained with hematoxylin. Positive slide was provided by the manufacture. Negative control sections were prepared with omission of the primary antibody. NF-κB appeared as brown nuclear and cytoplasmic reactions, whereas iNOS immunoreactivity appeared as a brown cytoplasmic reaction. The color image analysis system (a Leica Qwin 500 image analyzer connected to a Leica microscope, Schweiz, AG, Heerbrugg, CH-9435, Switzerland) was used to measure the optical density of NF-κB immune-expression in ten randomly selected fields and averaged per each section. The number of iNOS positive cells in ten randomly chosen high-power microscopic fields of the testicular sections was counted at a magnification of ×400 and averaged for each section. Four sections from four different rats were analyzed for each group.

### 2.10. Statistical Analysis

One-way analysis of variance (ANOVA, Tukey’s multiple comparison test) was performed using GraphPad Prism 6 statistic software (GraphPad Software, Inc., La Jolla, CA, USA) to compare among experimental groups. Data were normally distributed. Differences were considered statistically significant at *p* < 0.05. All data were expressed as means ± standard error of the mean (SEM).

## 3. Results

### 3.1. Effect of Vitamin D3 and/or Linagliptin on Body and Testicular Weights of Cisplatin-Treated Rats

As shown in Table 1, rats that underwent cisplatin exposure experienced reduced body and testes weights, compared to control rats (*p* < 0.001). Treatment with vitamin D3 or linagliptin tended to ameliorate the decline of body weight but did not reach statistical significance. However, the combined therapy slightly but significantly minimized the cisplatin-induced body weight loss (*p* < 0.05). Both vitamin D3 and linagliptin monotherapy significantly reversed the reduced testicular weight compared to cisplatin-treated rats (*p* < 0.05 and *p* < 0.001, respectively), but linagliptin exerted more remarkable effect on ameliorating the reduced testicular weight than vitamin D3 (*p* < 0.001). Notably, the combined therapy was much more effective in increasing testicular weight than vitamin D3 monotherapy only (*p* < 0.001), but comparable to the group treated with linagliptin.

### 3.2. Effect of Vitamin D3 and/or Linagliptin on Testicular Architecture in Cisplatin-Treated Rats

**Figure 1:**
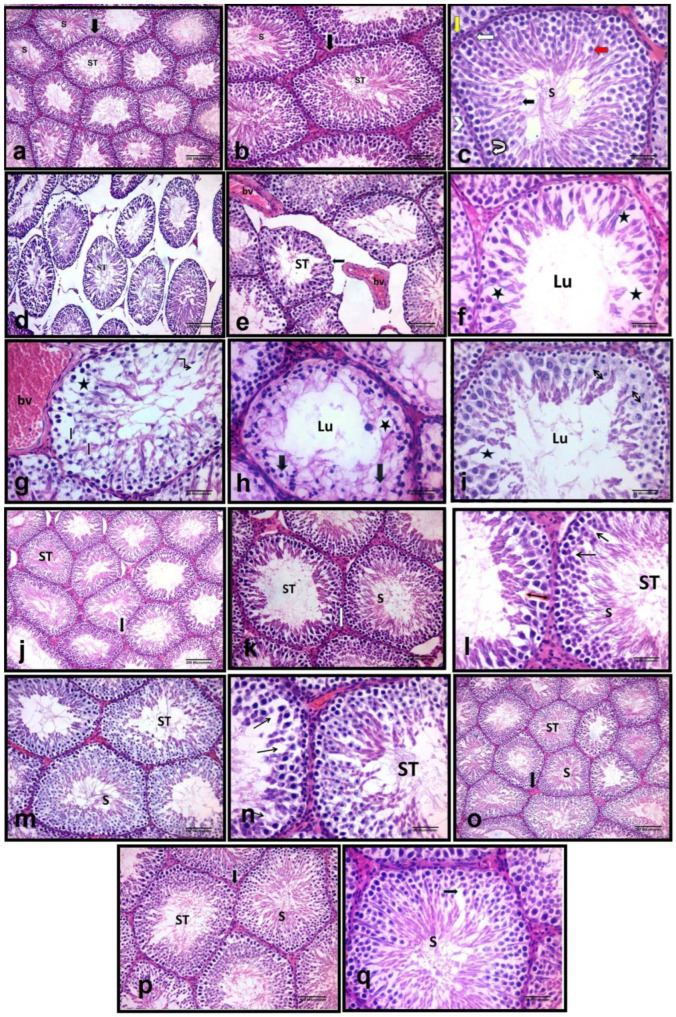
Representative photomicrographs of H&E-stained testicular sections from experimental groups. **CON group**: showed (**a**,**b**): ST = the seminiferous tubules; Arrow = the interstitial tissue; S = the sperms within the lumen of the seminiferous tubules. (**c**): Arrowhead = myoid cells; Straight white arrow = Sertoli cells; Yellow arrow = spermatogonia; Curved white arrow = primary spermatocytes; Red arrow = spermatid; S = the sperms; Black arrow: the lumen of the tubule. **CIS group:** showed (**d**,**e**) ST = the seminiferous tubules appear widely separated and irregular in shape; Black arrows = irregular basement membrane; bv = Large, dilated and congested blood vessel. (**f**) showed disorganized spermatogenic cells; black stars = wide spaces between the spermatogenic epithelium; Lu = wide lumen of the tubule with no sperm within it. (**g**,**h**): as the previous sections moreover, ˩ = vacuolated cytoplasm; black arrows = pyknotic small darkly stained nuclei; Black star = wide spaces between the spermatogenic cells, bv = dilated and congested blood vessel. (**i**): ↔ = The nuclei of some spermatocytes show division arrest; Black star = wide spaces between the spermatogenic cells; Lu = wide lumen of the tubules no sperm within it. **VD3 group** showed (**j**,**k**); ST = the seminiferous tubules appear closely packed, mostly rounded in shape; S = sperm; Arrows = the interstitial tissue. **(l):** Black arrow = spaces in between spermatogenic cells; Double head black arrow = decreased numbers of spermatogenic cells. **LINA group** showed (**m**,**n**): ST: the seminiferous tubules appear closely packed, mostly rounded in shape. Black arrows = spaces in between spermatogenic cells. **VD3+LINA group** showed (**o**,**p**): the histological structure of the seminiferous tubules appears nearly as that of the control group**.** (**q**): Black arrow: spaces in between spermatogenic cells **[H&E; (a**,**d**,**j**,**o) ×100 scale bar = 200 μm; (b**,**e**,**k**,**m**,**p) ×200 scale bar = 100 μm & (c**,**f**,**g**,**h**,**i**,**l**,**n**,**q) ×400 scale bar = 50 μm]**.

As shown in Figure 1, examination of H&E-stained sections of control group revealed that the testes were formed of multiple closely packed seminiferous tubules that were rounded and surrounded by a thin regular basement membrane. Seminiferous tubules were lined by stratified epithelium containing spermatogenic cells and Sertoli cells. Sertoli cells were observed at intervals between spermatogenic cells, characterized by their large triangular or ovoid vesicular nuclei with prominent nucleoli and resting on their basement membrane. Spermatogenic cells included spermatogonia, primary and secondary spermatocytes, spermatids, and finally mature sperm. The basal layer of the germinal epithelial cells was formed of a row of spermatogonia. Spermatogonia were small cells resting on the basement membranes. Primary spermatocytes appeared as the largest spermatogenic cells and were commonly observed in the sections. Secondary spermatocytes were small cells and rarely detected due to rapid division. Spermatids appeared as small rounded cells with rounded central vesicular nuclei situated near the lumen. Sperms were detected with their elongated nuclei attached by their heads to the Sertoli cells. They filled the lumen of seminiferous tubules. The interstitial space between the seminiferous tubules was formed of vascular connective tissue. It contained blood capillaries and groups of interstitial cells of Leydig. These cells were arranged either singly or in clusters (Figure 1a–c). Testicular sections from cisplatin-treated group revealed a loss of the normal architecture of the seminiferous tubules. They appeared in irregular variable sizes and were widely separated. The spermatogenic cells were disorganized and the basement membranes appeared irregular. Areas of cellular loss with multiple intercellular vacuoles were also observed in between the spermatogenic cells. In most tubules, the spermatogenic cells decreased in number to a few layers. Consequently, the lumen of the tubules appeared wide and few or no sperm were observed. The spermatogenic cells had vacuolated cytoplasm and pyknotic, small, darkly stained nuclei. Increased interstitial space was observed with dilated congested blood vessel. Arrest of mitotic division of germ cells was also seen (Figure 1d–i). Testicular sections from vitamin D3-treated groups showed that the normal shape and architecture of some seminiferous tubules were restored. Many seminiferous tubules appeared closely packed, mostly rounded and surrounded by a thin regular basement membrane. Many spermatogenic cells appeared well organized, and sperm were observed in most of the tubules. However, wide spaces were detected between these spermatogenic cells (Figure 1j–l). Testicular sections from linagliptin-treated group showed that most of the seminiferous tubules restored their normal shape and appeared multiple, closely packed, mostly rounded and surrounded by a thin regular basement membrane. Spermatogenic cells appeared well organized, and sperm were observed in most of the tubules. However, some spaces were still detected between these spermatogenic cells (Figure 1m,n). Testicular sections from the combined vitamin D3 and linagliptin-treated group revealed apparent normal seminiferous tubules. The tubules restored regular shape, spermatogenic layers were well organized, and sperm were observed in most of the tubules (Figure 1o–q).

**Figure 2:**
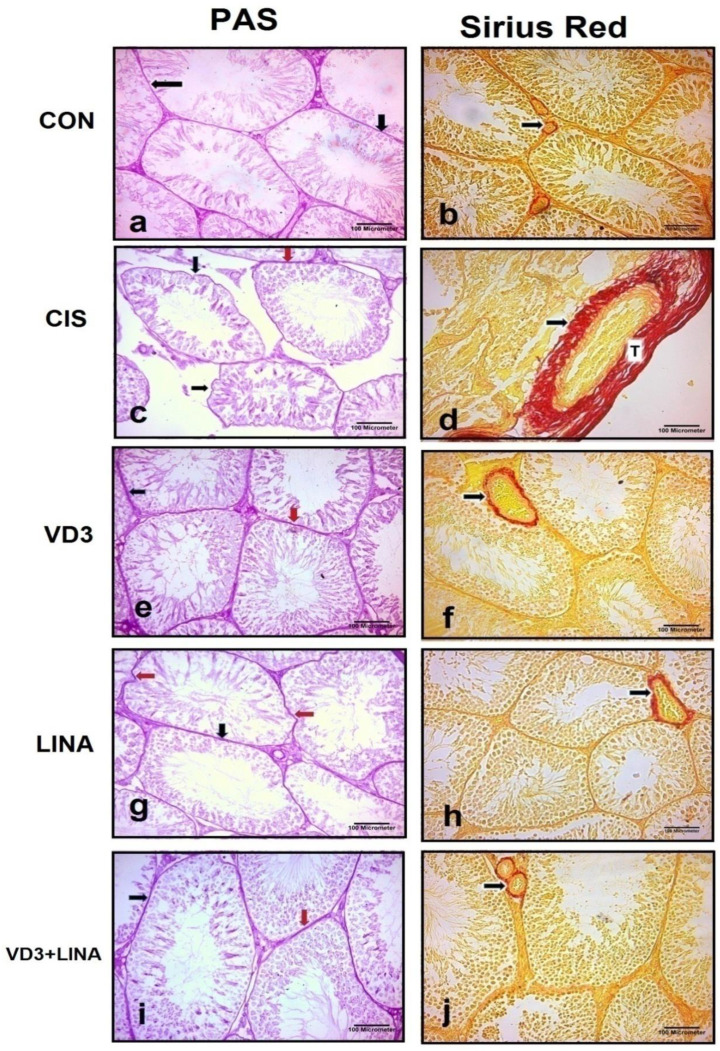
Representative photomicrographs of PAS-(Left panel) and Sirius red (Right panel)-stained testicular sections from experimental groups. **CON group**: showed (**a**): Black arrows = thin basement membranes surrounding seminiferous tubules (**b**): Black arrow = minimal collagen fibers in basal lamina around the seminiferous tubule and blood vessel’s wall in interstitial tissue; **CIS group** showed (**c**): Red arrows = thick basement membrane; Black arrows = irregular basement membrane surrounding the seminiferous tubules. (**d**): Black arrows = excessive collagen fibers depositing around the blood vessel’s wall; T = the tunicaalbuginea. **VD3 group** showed (**e**): Red arrow = thin regular basement membrane surrounding seminiferous tubules; Black arrow = thick basement membrane. (**f**): Arrow = moderate deposition of collagen fibers around blood vessel’s wall in interstitial tissue. **LINA group** showed (**g**): Black arrows = thin regular basement membrane surrounding seminiferous tubules; Red arrows = irregular parts of basement membrane. (**h**): Arrows = moderate deposition of collagen fibers around blood vessel’s wall in interstitial tissue as compared to CIS group; **VD3+LINA group** showed (**i**): Black arrow = thin regular basement membranes of the tubules more or less as that of CON group; Red arrow = thick parts of basement membrane. (**j**): Arrow: minimal (fine) collagen fibers around the blood vessel’s wall in interstitial tissue more or less as that of CON group. **(PAS and Sirius Red stain;** **×200, scale bar = 100 μm).**

As shown in Figure 2 left panel, the basement membrane reacted positively with PAS and appeared as a pink color in the stained sections. PAS-stained testicular sections from the control group showed clear, thin and regular basement membranes around the seminiferous tubules. There was an increase in PAS positive reaction with thickened and irregular basement membrane in cisplatin-treated group compared with control group. Sections from vitamin D3- or linagliptin-treated groups showed clear, positive PAS-reaction and thin basement membranes around the seminiferous tubules, although some parts of the basement membrane appeared thick or irregular. In combined therapy-treated group, PAS-stained sections revealed clear, positive PAS-reaction with thin and regular basement membranes around the seminiferous tubules.

As shown in Figure 2 right panel, Sirius red-stained testicular sections from the control group showed minimal collagen fibers (stained red) in basal lamina of seminiferous tubules and interstitial blood vessel’s wall. Testicular sections from cisplatin-treated group revealed an excessive amount of the collagen fibers around the wall of the interstitial blood vessels. Such a finding decreased in testicular sections from vitamin D3- or linagliptin-treated groups. In combined therapy-treated group, fine collagen fibers were seen around the interstitial blood vessel’s wall similarly to that of the control group.

As shown in Table 2, morphometric analyses showed significant reduction in seminiferous tubules diameter and germinal epithelial height, with a marked increase in the basement membrane thickness and area percentage of fibrotic area in the cisplatin-treated rats, compared to control group (*p* < 0.001). Treatment with vitamin D3 or linagliptin significantly increased seminiferous tubules diameter and germinal epithelial height (*p* < 0.001), while reducing the thickness of basement membrane and the fibrotic area (*p* < 0.001). Co-treatment of vitamin D3 with linagliptin exerted more remarkable effect than either monotherapy and effectively normalized the cisplatin-induced changes.

### 3.3. Effect of Vitamin D3 and/or Linagliptin on Spermatogenesis and Somatic Populations in Cisplatin-Treated Rats

As shown in Table 3, spermatogenesis in testicular tissue was assessed using Johnsen score (JS). There was a significant reduction in JS values in testicular sections of cisplatin-treated rats, compared to control group (*p* < 0.001), indicating disrupted spermatogenesis. Treatment of cisplatin-exposed rats with vitamin D3 and/or linagliptin significantly increase JS values (*p* < 0.001). The combined therapy effectively normalized the JS value. On the other hand, no significant differences either in numbers of Sertoli or Leydig cells were detected among different groups of rats.

### 3.4. Effect of Vitamin D3 and/or Linagliptin on Gonadal Hormone Levels in Cisplatin-Treated Rats

As shown in Table 4, both serum and testicular testosterone levels decreased significantly following cisplatin injection, with respect to control rats (*p* < 0.001), whereas both individual therapies of vitamin D3 and linagliptin similarly induced significant increases in testosterone levels in serum and testicular tissue, compared to the untreated group (*p* < 0.01 and *p* < 0.001, respectively). The combination of vitamin D3 and linagliptin almost restored serum testosterone levels to those of control rats much better than either monotherapy (*p* < 0.001). On the other hand, the serum levels of LH did not statistically differ among the experimental groups of rats, which might rule out the likelihood of hypothalamic-pituitary dysfunction.

### 3.5. Effect of Vitamin D3 and/or Linagliptin on Expressions of Steroidogenesis-Related Genes in Testes of Cisplatin-Treated Rats

**Figure 3:**
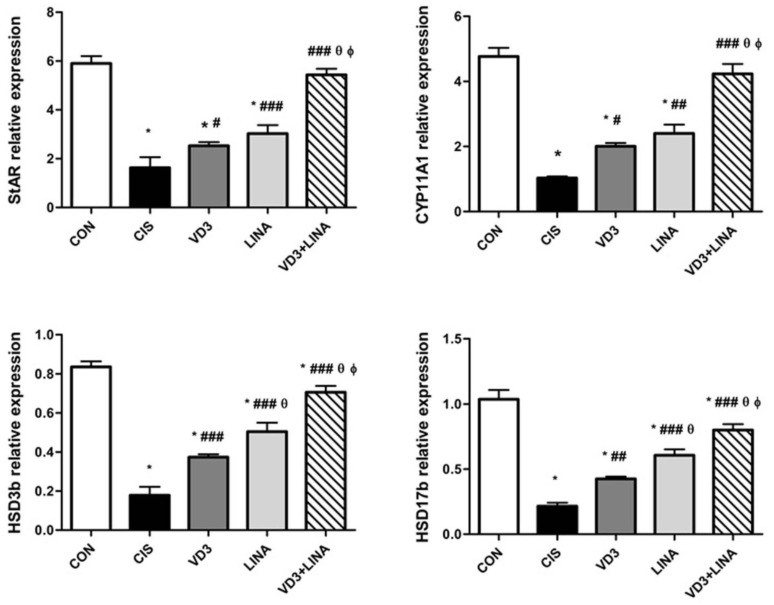
Effect of vitamin D3 and/or linagliptin on the relative expressions of steroidogenesis-related genes in testicular tissue of cisplatin-treated rats. Bars with error bars represent mean ± SEM (*n* = 6/group); * *p* < 0.001 vs. CON group; ^#^ *p* < 0.05, ^##^ *p* < 0.01, ^###^ *p* < 0.001 vs. CIS group; ^θ^ *p* < 0.001 vs. VD3 group; ^ϕ^ *p* < 0.001 vs. LINA group. StAR: Steroidogenic acute regulatory protein; CYP11A1: cytochrome P450 family 11, subfamily A1; HSD3b: 3 beta-hydroxysteroid dehydrogenase; HSD17b: 17-beta-hydroxysteroid dehydrogenase; CON group: control rats; CIS group: cisplatin-treated rats receiving drug vehicle; LINA group: cisplatin-treated rats receiving linagliptin therapy for 4 weeks; VD3 group: cisplatin-treated rats receiving vitamin D3 therapy for 4 weeks; VD3+LINA group: cisplatin-treated rats receiving combined therapy of linagliptin and vitamin D3 for 4 weeks.

As shown in Figure 3, the expressions of steroidogenic pathway genes in testicular tissue were measured by qRT-PCR. The StAR protein plays a pivotal role in cholesterol transport from the outer to the inner mitochondrial membrane which is the rate-limiting step in steroidogenesis. Biosynthesis of testosterone is catalyzed by two major types of enzymes named CYPs and HSDs [3]. There was a marked down-regulation in the gene expression of StARin testicular tissue of rats exposed to cisplatin as compared with the controls (*p* < 0.001). Cisplatin significantly down-regulated the mRNA expression of CYP11A1, HSD3b and HSD17b genes in testicular tissue of rats, with respect to control group (*p* < 0.001). Treatment with either vitamin D3 or linagliptin significantly increased the transcript levels of StAR, CYP11A1, HSD3b and HSD17b genes, in comparison to cisplatin-exposed rats. The positive effect of linagliptin therapy was comparable to that of vitamin D3 on the expression of steroidogenic markers, except HSD3b and HSD17b (*p* < 0.001). Co-administration of vitamin D3 with linagliptin almost restored steroidogenic markers, to greater extent than either monotherapy (*p* < 0.001).

### 3.6. Effect of Vitamin D3 and/or Linagliptin on Testicular Endoplasmic Reticulum Stress in Cisplatin-Treated Rats

**Figure 4:**
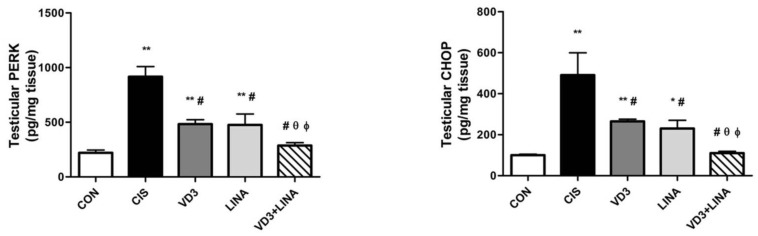
Effect of vitamin D3 and/or linagliptin on levels of ER stress-related proteins in testicular tissue of cisplatin-exposed rats. Bars with error bars represent means ± SEM, (*n* = 6); * *p* < 0.01, ** *p* < 0.001 vs. CON group; ^#^ *p* < 0.001 vs. CIS group; ^θ^ *p* < 0.01 vs. VD3 group; ^ϕ^ *p* < 0.01 vs. LINA group. PERK: protein kinase RNA-like ER kinase; CHOP: transcription factor C/EBP homologous protein; CON group: control rats; CIS group: cisplatin-treated rats receiving drug vehicle; LINA group: cisplatin-treated rats receiving linagliptin therapy for 4 weeks; VD3 group: cisplatin-treated rats receiving vitamin D3 therapy for 4 weeks; VD3+LINA group: cisplatin-treated rats receiving combined therapy of linagliptin and vitamin D3 for 4 weeks.

As shown in Figure 4, the levels of PERK and CHOP were assessed as well-established ER stress markers by ELISA. Cisplatin-exposed rats displayed significantly higher levels of PERK and CHOP in their testicular tissue than in control group (*p* < 0.001). Treatment with either vitamin D3 or linagliptin significantly and similarly attenuated the testicular levels of PERK and CHOP with respect to cisplatin-exposed rats receiving drug vehicle (*p* < 0.001). Co-administration of vitamin D3 with linagliptin induced more suppressive effect than either monotherapy regarding PERK and CHOP levels (*p* < 0.01). The combined therapy effectively restored the levels of ER stress markers to those of control rats.

### 3.7. Effect of Vitamin D3 and/or Linagliptin Therapy on NF-κB Expression in Testicular Tissue of Cisplatin-Treated Rats

**Figure 5:**
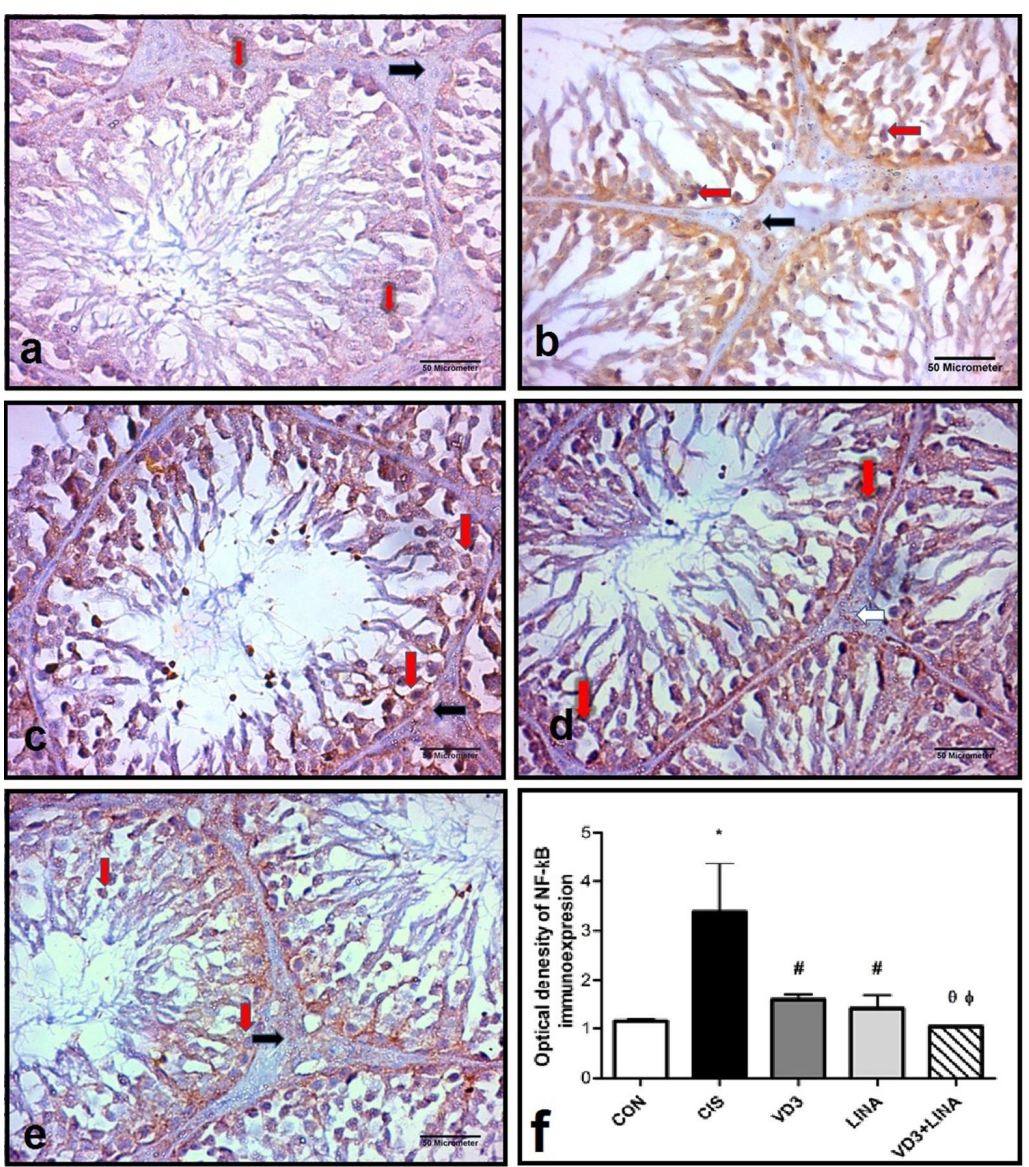
Immunohistochemical staining of NF-κB in testicular sections from experimental groups. NF-κB appeared as brown cytoplasmic and nuclear immunoreactions (avidin biotin peroxidase stain with Hx counter stain ×400, scale bar = 50 μm); (**a**): CON group showing weak nuclear and cytoplasmic immunoreaction for NF-κB spermatogenic cells (red arrows) and interstitial cells of Leydig (black arrow); (**b**): CIS group showing strong nuclear and cytoplasmic immunoreaction for NF-κB in spermatogenic cells (red arrows) and interstitial cells of Leydig (black arrows), as compared with that in the CON group; (**c**): VD3 group showing weak nuclear and cytoplasmic immunoreaction for NF-κB in in spermatogenic cells (red arrows) and interstitial cells of Leydig (black arrow); (**d**): LINA group showing weak nuclear and cytoplasmic immunoreaction for NF-κB in spermatogenic cells (red arrows) and interstitial cells of Leydig (black arrow); (**e**): VD3+LINA group showing minimal nuclear and cytoplasmic immunoreaction for NF-κB (arrows) in spermatogenic cells (red arrows) and interstitial cells of Leydig (black arrow) almost similar to that of the control group; (**f**): Quantification of NF-κB expression in testicular tissue. The optical density of NF-κB immune-expression was measured in ten randomly selected fields and averaged per each section. Bars with error bars represented mean ± SEM; * *p* < 0.001 vs. CON group; ^#^ *p* < 0.001 vs. CIS group; ^θ^ *p* < 0.05 vs. VD3 group; ^ϕ^ *p* < 0.05 vs. LINA group.

The immunohistochemical staining NF-κB was illustrated in Figure 5. Intense nuclear and cytoplasmic immunoreactivity of NF-κB was observed in spermatogenic and Leydig cells of cisplatin-treated group, while weak nuclear and cytoplasmic immunoreactivity of NF-κB was observed in spermatogenic and Leydig cells of CON, VD3, LINA and VD3+LINA groups. The optical density of NF-κB immunoexpression significantly increased in testicular tissue of cisplatin-treated rats, as compared to the control group (*p* < 0.001). Vitamin D3 and/or linagliptin effectively and similarly restored the immunoreactivity of NF-κB. The combined therapy exerted a superior effect to either monotherapy (*p* < 0.05).

### 3.8. Effect of Vitamin D3 and/or Linagliptin Therapy on Inflammatory Status in Testicular Tissue of Cisplatin-Treated Rats

**Figure 6:**
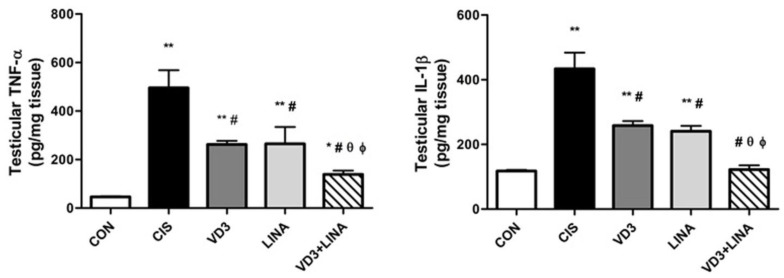
Effect of vitamin D3 and/or linagliptin on levels of inflammatory cytokines in testicular tissue of cisplatin-treated rats. Bars with error bars represent mean ± SEM (*n* = 6/group); * *p* < 0.05, ** *p* < 0.001 vs. CON group; ^#^ *p* < 0.001 vs. CIS group; ^θ^ *p* < 0.01 vs. VD3 group; ^ϕ^ *p* < 0.01 vs. LINA group. TNF-α: Tumor necrosis factor-alpha; IL-1β: Interleukin-1beta; CON group: control rats; CIS group: cisplatin-treated rats receiving drug vehicle; LINA group: cisplatin-treated rats receiving linagliptin therapy for 4 weeks; VD3 group: cisplatin-treated rats receiving vitamin D3 therapy for 4 weeks; VD3+LINA group: cisplatin-treated rats receiving combined therapy of linagliptin and vitamin D3 for 4 weeks.

As shown in Figure 6, there was a dramatic increase in the testicular levels of TNF-α and IL-1β in cisplatin-treated rats, compared with control group (*p* < 0.001). Treatment of cisplatin-exposed rats with vitamin D3 or linagliptin significantly suppressed the testicular levels of TNF-α and IL-1β (*p* < 0.001) to a similar extent, indicating the anti-inflammatory effect of both therapies. The combined regimen significantly reversed the augmented inflammatory response to a greater extent than either individual therapy (*p* < 0.01).

### 3.9. Effect of Vitamin D3 and/or Linagliptin Therapy on iNOS Expression in Testicular Tissue of Cisplatin-Treated Rats

**Figure 7:**
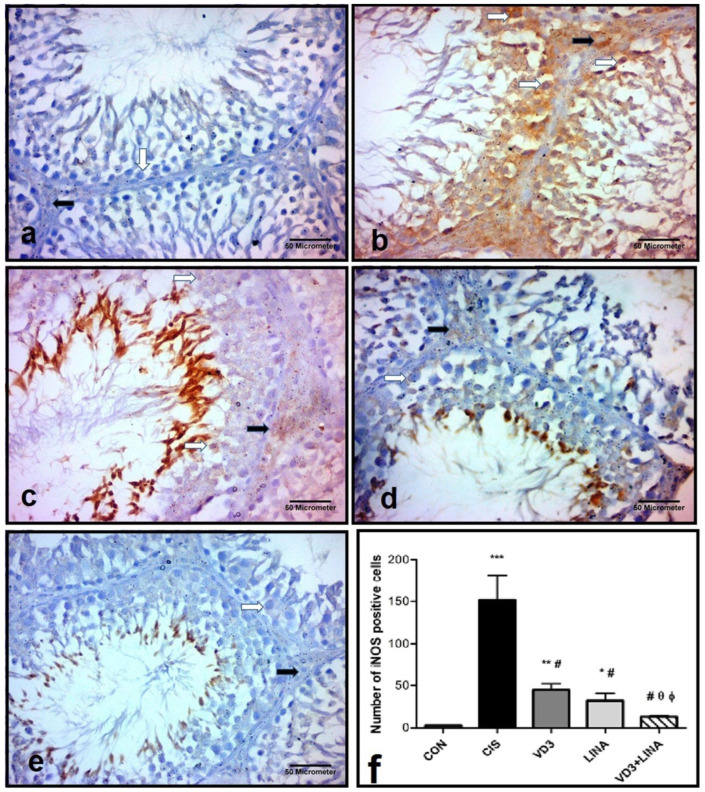
Immunohistochemical staining of iNOS in testicular sections from experimental groups. iNOS immunoreactivity appeared as brown cytoplasmic staining; (avidin biotin peroxidase stain with Hx counter stain x400, scale bar = 50 μm); (**a**): CON group showing scanty immunoexpression of iNOS within the spermatogenic cells (white arrows) and interstitial cells of Leydig (black arrow); (**b**): CIS group showing marked expression of iNOS within the spermatogenic cells (white arrows) and interstitial cells of Leydig (black arrow), compared to control group; (**c**): VD3 group showing decrease in the expression of iNOS within the spermatogenic cells (white arrows) and interstitial cells of Leydig (black arrow); (**d**): LINA group showing decrease in the expression of iNOS within the spermatogenic cells (white arrows) and interstitial cells of Leydig (black arrow); (**e**): VD3+LINA group showing apparent decrease in the expression of iNOS within the spermatogenic cells (white arrows) and interstitial cells of Leydig (black arrow); (**f**): Quantification of iNOS expression in testicular tissue. The number of iNOS positive cells was counted in ten randomly selected high-power microscopic fields and averaged per each animal. Bars with error bars represented mean ± SEM (*n* = 4); * *p* < 0.05, ** *p* < 0.01, *** *p* < 0.001 vs. CON group; ^#^ *p* < 0.001 vs. CIS group; ^θ^ *p* < 0.05 vs. VD3 group; ^ϕ^ *p* < 0.05 vs. LINA group.

The immunohistochemical staining iNOS was illustrated in Figure 7. A weak brown cytoplasmic immunoreactivity of iNOS was observed in spermatogenic and Leydig cells of CON, VD3, LINA and VD3+LINA groups, while a strong cytoplasmic immunoreactivity of iNOS was found in spermatogenic and Leydig cells of the cisplatin-treated group. The numbers of iNOS-positive cells dramatically increased in the CIS group, when compared to control group (*p* < 0.001). Treatment with either vitamin D3 or linagliptin significantly reduced the number of iNOS-positive cells (*p* < 0.001) to a similar extent. The combined therapy normalized the iNOS-positive cells and induced a much better effect than either monotherapy (*p* < 0.05).

## 4. Discussion

Testicular dysfunction and male infertility in consequence to cisplatin-based chemotherapy is still a medical concern in male cancer survivors [24,25]. Therefore, restoring steroidogenesis and spermatogenesis after cisplatin chemotherapy is of clinical importance. In the present study, rats that underwent cisplatin injection displayed decreased testicular weight. Testicular architecture was severely deteriorated with a reduction in seminiferous tubule diameter and epithelial height, but increased thickness of basement membrane and deposition of collagen fibers around the interstitial blood vessels in cisplatin-treated rats. In those rats, impaired spermatogenesis was mirrored from declined JS values and loss of gem cells. Furthermore, there was significantly lower testicular testosterone level in cisplatin-treated rats than control ones. Testosterone levels in the testicular tissue closely reflected those observed in the serum, in agreement with published studies [26,27]. The numbers of Leydig cells remained unaffected following cisplatin exposure; thus, the reduced testosterone levels appeared to result from the relative unresponsiveness of Leydig cells to LH in cisplatin-treated rats. The unchanged serum LH levels suggested that the decline in testosterone levels was not a consequence of hypothalamic-pituitary changes [28]. These findings together suggested that the reduced circulating and testicular testosterone were secondary to the aberrant steroidogenic machinery in Leydig cells.

The present study clearly unraveled the positive effect of vitamin D3, linagliptin and their combination on testicular steroidogenesis and testosterone biosynthesis after cisplatin injection. Herein, we found that vitamin D3 supplementation for four weeks significantly increased serum and testicular levels of testosterone, likely through up-regulation of steroidogenesis-related genes including StAR, CYP11A1, HSD3b and HSD17b in testicular tissue of cisplatin-exposed rats. The modulatory effect of vitamin D3 on testicular steroidogenesis has been depicted previously in both in vivo and in vitro experimental studies [29,30]. A recent report suggests that vitamin D3 promotes testosterone biosynthesis in Leydig cells, through activation of the insulin-like growth factor-1 (IGF-1) signaling pathway [31]. This effect might be mediated through VDR-dependent mechanisms, since the expression of VDR is widely distributed in testes [13], although we could not completely exclude the contribution of VDR-independent mechanisms. Further studies using VDR antagonist or a Vitamin D 25-hydroxylation inhibitor should be used to certify or deny our speculation.

We also found that linagliptin therapy for four weeks could increase the circulatory and intra-testicular testosterone, in consistence with our previous work [16]. Linagliptin therapy ameliorated the cisplatin-induced down-regulation of steroidogenesis-related genes, thereby stimulating the steroid hormone biosynthetic pathway in testicular tissue. These results suggested that vitamin D3 and linagliptin synergistically counteracted the suppressive effect of cisplatin on steroidogenesis through inducing the transcription of steroidogenic proteins and enzymes. Thereby, the combination of linagliptin with vitamin D3 could increase serum testosterone by directly stimulating Leydig cell to synthesize testosterone. These findings suggested that such therapeutic intervention might serve as a promising modality to restore testicular steroidogenesis following cisplatin therapy. Testosterone is crucial to ensure proper spermatogenesis and fertility by inducing spermatogonial proliferation and meiotic divisions leading to the production of spermatozoa [2]. Consistently, Leydig cell dysfunction and aberrant steroidogenesis seen in cisplatin-treated rats were accompanied with deteriorated spermatogenesis. Rats treated with either vitamin D3 and/or linagliptin displayed a wider diameter of seminiferous tubules, a thicker germinal epithelium and more reduced fibrosis than cisplatin-treated controls. Both therapies, either individually or in combined form, reversed the declined JS values reflecting the promotion of germ cells repopulation. These data suggested restoration of testicular architecture and enhancement of the spermatogenic activity after linagliptin/vitamin D3 therapies, in parallel with previous reports [32,33].

ER stress has been shown to impair Leydig cell steroidogenic function and inhibit testosterone production in several pathological conditions [34,35,36]. Herein, the up-regulation of PERK and CHOP in testicular tissue of cisplatin-treated rats depicted the activation of UPR and ER stress, in agreement with published studies [11,37]. ER stress is triggered by the accumulation of unfolded or misfolded proteins caused by reactive oxygen species, iron imbalance, calcium leakage, protein overload, and/or hypoxia. Activation of PERK, as a major UPR sensor, down-regulates the global protein synthesis by phosphorylating the eIF2α, a translation initiation factor, causing translational shut-down for multiple proteins, including steroidogenic enzymes [38]. Consequently, it seemed plausible to assume that the down-regulation of testicular steroidogenesis-related genes in cisplatin-treated rats might be mediated, at least in part, to the activation of PERK.

The three ER stress sensors can amplify the inflammatory burden by activating NF-κB via different mechanisms. Under basal conditions, NF-κB is sequestered in the cytoplasm in a complex with inhibitor kappa B, (IκBα), which prevents the nuclear translocation of NF-κB. Canonical activation of NF-κB involves phosphorylation of IκBα by inhibitor kappa B kinase (IKK), followed by proteosomal degradation of IkBα [39]. PERK has been shown to phosphorylate the eIF2α, thereby repressing the synthesis of the free unbound IκBα, enabling the NF-κB to translocate into the nucleus. This can trigger a persistent inflammatory response by up-regulating the expression of genes coding for pro-inflammatory cytokines such as TNF-α and IL-1β. Supportively, PERK inhibitors have been shown to exert a suppressive effect on NF-κB target genes [40]. A previous study has underlined CHOP as a transcription factor essential for NF-κB activation, thus linking ER stress to inflammation and cell death [41]. Consistent with the findings cited above, vitamin D3 supplementation was found to down-regulate the expression of PERK and CHOP, suggesting the modulatory effect of vitamin D3 on UPR and ER stress in testicular tissue. Relevant to our results, vitamin D3 has been shown to inhibit the aberrant activation of ER stress in the hippocampus of diabetic rats [42]. Similarly, the expressions of ER stress markers in testicular tissue of cisplatin-exposed rats were suppressed by linagliptin, emphasizing that the combined therapy exerted more inhibitory effect on ER stress than either monotherapy. In consistence with our findings, linagliptin exerted an inhibitory effect on ER stress in cisplatin-induced neurotoxicity [43]. Furthermore, sitagliptin, another DPP-4 inhibitor, has been reported to mitigate ER stress in diabetic rat testis [44]. Collectively, these results suggest that mitigation of ER stress by vitamin D3 and linagliptin might be involved in their therapeutic potential against cisplatin-induced testicular toxicity

NF-κB is a transcription factor considered to be a master regulator of the immune and stress responses. In the rat testis, the NF-κB complex is constitutively expressed in Sertoli cells, germ cells and Leydig cells [45]. There is increasing evidence highlighting the NF-κB as a key candidate for the regulation of male germ cell apoptosis owing to its evident role in cell proliferation and survival. Pharmacological inhibition of testicular NF-κB activation effectively suppressed the germ cell loss [46]. Accordingly, the increased cytoplasmic and nuclear expression of NF-κB in spermatogenic cells implied the nuclear translocation of NF-κB and transcriptional activation of stress genes. Thus, the activation of NF-κB signaling pathway with the subsequent increase in the inflammatory cytokines might underlie the perturbations in spermatogenesis and loss of gem cells seen in cisplatin-treated rats. A similar situation was found in Leydig cells, where intense cytoplasmic and nuclear staining for NF-κB was found. Given that NF-κB has been shown to mediate glucocorticoid-induced Leydig cell apoptosis [47], the activation of NF-κB might participate in the steroidogenic dysfunction seen in the testis of rats that underwent cisplatin exposure. Intriguingly, vitamin D3 and linagliptin synergistically suppressed the NF-κB activation in testicular tissue. Relevant to our findings, a previous study has shown that vitamin D3 supplementation can improve testicular function in diabetic rats via blocking NF-κB signaling pathway [48]. Likewise, linagliptin has been reported to suppress NF-κB activation in cardiac tissue and serum inflammatory cytokine levels in type II diabetic mice with induced sepsis [49].

There is emerging evidence demonstrating a key role of iNOS in autocrine or paracrine regulation of the testicular vasculature, steroidogenesis and spermatogenesis in normal and pathological conditions. The iNOS is up-regulated in testicular tissues in response to inflammatory mediators and cytokines, and produces relatively larger amounts of nitric oxide (NO) than the other two isoforms; endothelial and neuronal NOS [50]. An earlier study has shown that the up-regulation of iNOS could compromise testicular steroidogenesis, presumably through its direct action on HSD3b and CYP17A1 [51]. Furthermore, it has been demonstrated that the increased iNOS expression is linked with declined serum testosterone and down-regulation of StAR expression in the testis of male mouse after exposure to bisphenol A (BPA) [52]. Our findings revealed intense expression of iNOS in Leydig and spermatogenic cells in cisplatin-treated rats, which appeared to be implicated in the compromised steroidogenesis and spermatogenesis seen in those rats. Vitamin D3 and linagliptin exerted a suppressive effect on cisplatin-induced up-regulation of iNOS expression in testicular tissue, to a similar extent. The modulatory effect of these drugs on iNOS expression appeared to be involved in their favorable effects on steroidogenesis and spermatogenesis. Our findings largely agree with a previous study showing that linagliptin can suppress the renal expression of NF-κB and iNOS in a rat model of acute kidney injury [53]. Collectively, these results might shed light on the therapeutic benefits of the combined linagliptin and vitamin D3 therapy for male patients under cisplatin chemotherapy to restore the deficiency of testicular testosterone and ameliorate the detrimental effects of cisplatin on testicular homeostasis, which consequently preserve male fertility.

## 5. Conclusions

The current study demonstrated for the first time that linagliptin and vitamin D3 synergistically could restore testicular steroidogenesis and spermatogenesis in cisplatin-treated rats, at least in part, through suppression of ER stress and NF-kB/iNOS axis. The present findings highlight the combination of linagliptin and vitamin D3 as a promising therapeutic modality to alleviate cisplatin-induced testicular toxicity in rats. These findings might hold the promise to restore male fertility following cisplatin chemotherapy, which could broaden the therapeutic spectrum of these drugs. Further investigations should be conducted for clinical translation of the present results.

## Figures and Tables

**Table 1:** Effect of vitamin D3 and/or linagliptin on body and testes weights in cisplatin-treated rats.

	Initial Body Weight (g)	Final Body Weight (g)	Total Testicular Weight (g)
**CON**	231.8 ± 4.12	246.3 ± 5.58	2.83 ± 0.026
**CIS**	226.3 ± 4.88	188.8 ± 7.07 **	2.08 ± 0.05 **
**VD3**	226.3 ± 3.19	199.0 ± 3.27 **	2.33 ± 0.032 **^,#^
**LINA**	235.0 ± 4.29	206.8 ± 3. 21 **	2.63 ± 0.035 *^,##,^^θ^
**VD3+LINA**	236.8 ± 2.90	210.3 ± 5.19 **^,#^	2.65 ± 0.05 *^,##,^^θ^

Values represent mean ± SEM (*n* = 6/group); * *p* < 0.05, ** *p* < 0.001 vs. CON group; ^#^ *p* < 0.05, ^##^ *p*< 0.001 vs. CIS group; ^θ^ *p* < 0.001 vs. VD3 group. CON group: control rats; CIS group: cisplatin-treated rats receiving drug vehicle; LINA group: cisplatin-treated rats receiving linagliptin therapy for 4 weeks; VD3 group: cisplatin-treated rats receiving vitamin D3 therapy for 4 weeks; VD3+LINA group: cisplatin-treated rats receiving combined therapy of linagliptin and vitamin D3 for 4 weeks.

**Table 2:** Effect of vitamin D3 and/or linagliptin on morphometric parameters of seminiferous tubules in cisplatin-treated rats.

	Seminiferous Tubules Diameter (μm)	Germinal Epithelial Height (μm)	Basement Membrane Thickness (μm)	Fibrotic Area %
**CON**	430.2 ± 11.9	115.8 ± 3.6	2.37 ± 0.25	2.24 ± 0.24
**CIS**	246.5 ± 10.3 **	58 ± 2.5 **	16.6 ± 1.6 **	18.9 ± 1.05 **
**VD3**	331.3 ± 11.6 **^,#^	80.6 ± 2.7 **	6.22 ± 0.54 *^,#^	6.58 ± 0.69 **^,#^
**LINA**	380.4 ± 8.62 *^,#,^^θ^	92.7 ± 2.3 **^,^^θ^	5.92 ± 0.45 *^,#^	5.49 ± 0.26 *^,#^
**VD3+LINA**	427.4 ± 11.8 ^#,^^θθ,ϕ^	110 ± 2.1 ^#,^^θθ,ϕ^	2.46 ± 0.31 ^#,^^θ,ϕ^	2.82 ± 0.44 ^#,^^θ,ϕ^

Values represent mean ± SEM; Four testicular slides from different four rats were analyzed in each group; * *p* < 0.05, ** *p* < 0.001 vs. CON group; ^#^ *p* < 0.001 vs. CIS group; ^θ^ *p* < 0.05, ^θθ^ *p* < 0.001 vs. VD3 group; ^ϕ^ *p* < 0.05 vs. LINA group. CON group: control rats; CIS group: cisplatin-treated rats receiving drug vehicle; LINA group: cisplatin-treated rats receiving linagliptin therapy for 4 weeks; VD3 group: cisplatin-treated rats receiving vitamin D3 therapy for 4 weeks; VD3+LINA group: cisplatin-treated rats receiving combined therapy of linagliptin and vitamin D3 for 4 weeks.

**Table 3:** Effect of vitamin D3 and/or linagliptin on spermatogenesis and somatic population in testes of cisplatin-treated rats.

	Johnsen Score (JS) Per Tubule	No. of Sertoli Cells Per Tubule	No. of Leydig Cells Per Field
**CON**	8.95 ± 0.19	13.27 ± 0.49	16.90 ± 0.53
**CIS**	3.70 ± 0.31 **	12.91 ± 0.48	14.10 ± 1.03
**VD3**	7.70 ± 0.32 *^,#^	13.09 ± 0.89	15.70 ± 0.92
**LINA**	7.55 ± 0.25 *^,#^	13.0 ± 0.50	16.70 ± 0.58
**VD3+LINA**	8.40 ± 0.19 ^#^	13.18 ± 0.57	16.70 ± 0.59

Values represent mean ± SEM; Four H&E-stained testicular slides from different four rats were analyzed in each group; * *p* < 0.05, ** *p* < 0.001 vs. CON group; ^#^ *p* < 0.001 vs. CIS group. CON group: control rats; CIS group: cisplatin-treated rats receiving drug vehicle; LINA group: cisplatin-treated rats receiving linagliptin therapy for 4 weeks; VD3 group: cisplatin-treated rats receiving vitamin D3 therapy for 4 weeks; VD3+LINA group: cisplatin-treated rats receiving combined therapy of linagliptin and vitamin D3 for 4 weeks.

**Table 4:** Effect of vitamin D3 and/or linagliptin on gonadal hormone levels in cisplatin-treated rats.

	Serum LH Levels (ng/mL)	Serum Testosterone Levels (ng/mL)	Testicular Testosterone Levels (ng/g Tissue)
**CON**	90.83 ± 5.83	3.29 ± 0.19	16.81 ± 0.21
**CIS**	82.17 ± 3.05	0.81 ± 0.015 **	4.05 ± 0.47 **
**VD3**	82.67 ± 3.69	1.57 ± 0.20 **^,#^	6.60 ± 0.54 **^,#^
**LINA**	87.00 ± 6.16	1.90 ± 0.17 **^,##^	7.80 ± 0.41 **^,##^
**VD3+LINA**	87.17 ± 5.68	3.02 ± 0.23 ^###,^^θ,ϕ^	13.8 ± 1.07 *^,###,^^θ,ϕ^

Values represent mean ± SEM (*n* = 6/group); * *p* < 0.01, ** *p* < 0.001 vs. CON group; ^#^ *p* < 0.05, ^##^ *p* < 0.01, ^###^ *p* < 0.001 vs. CIS group; ^θ^ *p* < 0.001 vs. VD3 group; ^ϕ^ *p* < 0.001 vs. LINA group. LH: luteinizing hormone; CON group: control rats; CIS group: cisplatin-treated rats receiving drug vehicle; LINA group: cisplatin-treated rats receiving linagliptin therapy for 4 weeks; VD3 group: cisplatin-treated rats receiving vitamin D3 therapy for 4 weeks; VD3+LINA group: cisplatin-treated rats receiving combined therapy of linagliptin and vitamin D3 for 4 weeks.

## Data Availability

The datasets generated and/or analyzed during the current study are available upon reasonable request from the corresponding author.

## Sample Availability

Samples of the compounds are not available from the authors.
